# Army Ants as Research and Collection Tools

**DOI:** 10.1673/031.008.7101

**Published:** 2008-11-14

**Authors:** Adrian A. Smith, Kevin L. Haight

**Affiliations:** School of Life Sciences, Arizona State University, Tempe, AZ 85287-4501 USA

**Keywords:** *Neivamyrmex nigrescens*, *Aphaenogstercockerelli*, nest evacuation, absconding

## Abstract

Ants that fall prey to the raids of army ants commonly respond by evacuating their nests. This documented behavior has been underexploited by researchers as an efficient research tool. This study focuses on the evacuation response of the southwestern desert ant *Aphaenogaster cockerelli* André (Hymenoptera: Formicidae) to the army ant *Newamyrmex nigrescens* Cresson. It is shown that army ants can be used to collect mature colonies of ants. The applicability of this tool to ecologically meaningful areas of research is discussed.

## Introduction

A common defense response of ants to army-ant raids is nest evacuation. The rapid fleeing from the nest of workers with brood, sometimes in the company of the queen, is a well documented response of preyed-upon ant species throughout the New World (Schneirla 1971). The army ant *Neivamyrmex nigrescens* Cresson (Hymenoptera: Formicidae), which is distributed throughout the southwestern United States, is a particularly well studied species in which nest evacuation is a common response of their prey. Several species of ants across multiple genera are raided by this ant. The reported list of preyed-upon genera which are known to evacuate workers and brood from their nests in response to *N*. *nigrescens* includes: *Pheidole* (Droual 1983, 1984), *Trachymyrmex* (Topoff et al. 1980), *Camponotus* (LaMon and Topoff 1981) and *Aphaenogaster* (McDonald and Topoff 1986). Species in which the full colony (workers with brood and queen) evacuates in response to *N*. *nigrescens* include *Camponotus festinatus* (LaMon and Topoff 1981), *Aphaenogaster* (=*Novomessor*) *albisetosa* and *Aphaenogaster cockerelli* (McDonald and Topoff 1986).

Researchers documenting nest evacuation responses have successfully induced evacuations by introducing a relatively small number (100–150) of army ants to the targeted colony (McDonald and Topoff 1986; LaMon and Topoff 1981; Droual 1983, 1984). Although triggering an evacuation has been proven to be simple, this behavior has been poorly exploited in other areas of myrmecological research. A study by Helms and Rissing (1990) utilizes the evacuation response of *Pheidole* species to census alate production and is, to our knowledge, the lone example of utilizing army ants as a research tool. It is surprising that in the twenty years since nest evacuation behavior has been thoroughly documented that this method of extracting ants from the ground has not been more generally applied as a field research tool. In this methodological paper the evacuation response of *A. cockerelli* to *N*. *nigrescens* is demonstrated. The benefits of exploiting this behavior as a means for collecting mature colonies and its research applications are discussed.

## Methods and Results

*Aphaenogaster cockerelli* occurs in intermountain plains throughout the southwestern United States and into Mexico (Johnson 2000). Colonies are monogynous and mature colonies are polydomous, with 2–5 distinct nests (Hölldobler and Carlin 1989). For this study colonies were collected primarily from the Chihuahuan desert between Portal, Arizona and Rodeo, New Mexico from the end of August through the beginning of October, 2006 and in August, 2007 (N= 30). Collections were also made 12 km east of Apache Junction, Arizona in August, 2006 (N = 2). Three different collection methods were attempted: physical excavation, flood-triggered nest evacuation, and army-ant triggered nest evacuation.

Physical excavation was attempted on *A. cockerelli* colonies with small (∼ 15 cm diameter) nest mounds. Five days (45 hours) were spent on the excavation of 14 colonies; resulting in the collection of a total of four queenright colonies of a few hundred workers. The majority (n=10) of excavations yielded only workers and brood. Excavations frequently went one meter deep, and the queens that were collected were probably caught because they happened to be located near the surface. Single nest excavations, dug using picks and shovels, took approximately two to three hours.

A total of six colonies were flooded. The evacuation of brood was triggered after approximately 1.9 liters of water was poured into the nest entrance. Incrementally streaming water into the nest entrance gradually induced workers with brood to evacuate to a neighboring satellite nest but never resulted in the evacuation of the queen. After two to three hours of incremental flooding, the number of workers exiting the nest carrying brood diminished to zero.

The third method used workers of the army ant, *N*. *nigrescens*, to induce evacuation of *A. cockerelli* nests. Due to both *N. nigrescens* and *A. cockerelli* being crepuscular/nocturnal ants the collections of both species were done between 1800 hours and 0100 hours. *N. nigrescens* workers were collected from raiding columns. In a total of four collection trips, raiding columns of *N. nigrescens* were encountered by randomly walking and scanning the ground for raiding columns. To collect *A. cockerelli*, groups of 100–150 army ants where aspirated into 50 ml centrifuge tubes, shaken into ball-like masses, and poured directly into the entrances of *A. cockerelli* nests. Evacuation response was nearly instantaneous. Within fifteen seconds a steady stream of workers were running out of the nest (Video 1). Within thirty seconds to one minute workers were exiting carrying brood. Additional army ants were not introduced unless the rate of brood exiting the nest diminished significantly or workers reentered the nest with brood. Queens usually exited, or were carried, out of the nests after the first 150–300 army ants were introduced (Video 2). On some occasions when large nests (∼5,000–6,000 workers) were evacuated the queen appeared only after more than 300 army ants had been added. On average three colonies were collected per hour of work. A total of 28 queenright colonies with an estimated average size of two to three thousand workers were collected from two field sites using this method.

**Figure 1.**
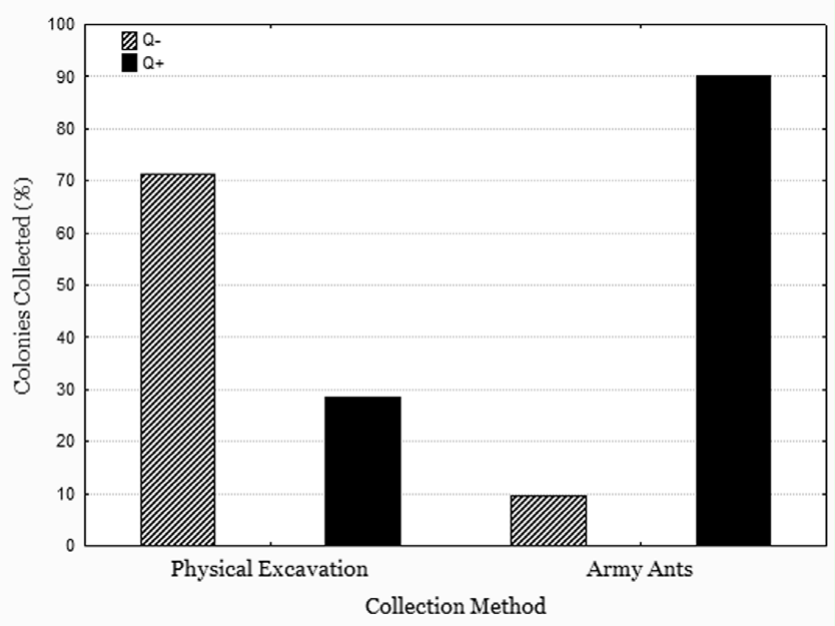
Collection of *Aphaenogaster cockerelli* colonies by physical excavation (n=14) and utilizing *Neivamyrmex nigrescens* ants (n=31). Using army ants proves to be a more effective method than physical excavation for capturing queenright (Q+) colonies (2-tailed Fisher exact test, p<0.0001). Queenless (Q-) colony collection represents both nests without a queen and failed collections where the queen (if present) could not be captured.

Queenright colonies of *A. cockerelli* could be collected much more effectively using army ants (Figure 1). Physical excavation yielded a single queenright colony per 11.25 hours of work, while a single queenright colony could be caught in just twenty minutes of work using army ants.

**Video 1.**
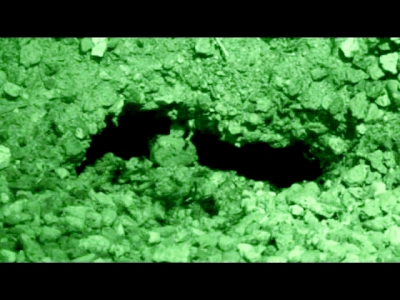
Worker nest evacuation response of *Aphaenogaster cockerelli* triggered by placing 150 *Neivamyrmex nigrescens* workers at the pictured nest entrance. **View this video online at** http://www.insectscience.org/8.71/ref/video1.html

**Video 2.**
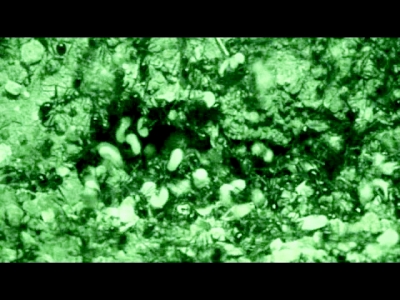
*Aphaenogaster cockerelli* workers evacuating carrying brood in response to the introduction of *Neivamyrmex nigrescens* workers, followed by the evacuation and collection of the queen. Video slowed to half-speed. **View this video online at** http://www.insectscience.org/8.71/ref/video2.html

## Discussion

Utilizing the nest-evacuation response of *A. cockerelli* when collecting mature colonies proves to be the most efficient means of colony collection. Not only is the method much faster than all others tried, but it also allowed the collection of large mature nests. Excavating small nests of a few hundred workers required two to three hours of work, whereas when using *N*. *nigrescens* as a collection tool collection of a queenright colony with 4,000 workers took approximately twenty minutes.

Knowledge of the natural behavioral patterns of these two ant species proves to be essential when undertaking field collections. The army ant *N*. *nigrescens* forages at night when *A. cockerelli* is most active and has been observed to preferentially raid *A. cockerelli* nests in late summer and early fall when conditions are dryer and other prey species (*Pheidole*) become less active (Mirenda et al. 1980). The collections described here were done under these conditions. The same species of army ant is known to prey on several other co-occurring ant species, as listed above, and elicit a full colony evacuation (with queen) in at least two other species (Lamon and Topoff 1981; McDonald and Topoff 1986). This common evacuation response of several ant genera to this species of army ant suggests that this approach to collection could be an effective method for the collection of other ant species that are preyed upon by army ants.

Beyond collecting mature colonies from the field, the evacuation responses of preyed-upon species can be exploited in other areas of research. After evacuation, species from several genera have been documented to reenter the original nest (*Phiedole*: Mirenda et al. 1980; Droual 1984. *Trachymyrmex*: LaPolla et al. 2002. *Aphaenogaster*: personal observations). By triggering evacuations and collecting the evacuating workers and brood, sociometric data can be easily gathered. Since the colony will reenter the nest colonies can be non-destructively assessed multiple times throughout development, providing valuable descriptive life history data (Tschinkel 1991). The question of the impact of such repeated evacuations on the colony remains to be investigated.

Promising studies of queen turnover or replacement are possible in systems where queens evacuate with their colony and nests are re-inhabited. Three monogynous species that are known to evacuate with the queen in response to *N*. *nigrescens* include *Camponotus festinatus*, *Aphaenogaster albisetosa* and *A. cockerelli*. Queen replacement in orphaned monogynous species has been shown to occur both by adoption of foreign queens and daughter queens from within the nest (Sanetra and Crozier 2002; Tschinkel 1996; DeHeer and Tschinkel 1998). Studies of genetic colony kin structure predict queen replacement in monogynous species to be an important occurrence in the life cycle of a colony (Heinze and Keller 2000). Using army ants to remove the queen without damaging the nest structure or the worker population is quite possible and would be a worthwhile method to study the behavior and reproductive efforts of orphaned colonies in their natural environment.

Finally, studies of interspecific competition often involve the removal of one of the competing species (King and Tschinkel 2006; LeBrun et al. 2007). Removing a targeted species in an ecologically non-destructive and responsible way is of great importance to these studies and has taken considerable effort (Tschinkel and King 2007). Using army ants to non-destructively remove a competitive species is an as-of-yet unused but promising method. For example, *A. cockerelli* is a species that co-occurs with other seed harvesting ants such as *Pogonomyrmex barbotus* and *Pogonomyrmex rugosus* (Johnson RA 2000; Hölldobler et al. 1978), and is likely to compete with these species along with other smaller omnivorous ant species such as *Solenopsis xyloni and Monomorium minimum* (Hölldobler et al. 1978). *A. cockerelli* is therefore a prime example of a competitive species that can easily be removed from an ecosystem without harming the habitat or co-occurring fauna in the process.

Utilizing army ants to induce nest evacuations in prey species is a promising, and often overlooked tool. The applicability of this method seems to be far reaching if the faunal system including *N. nigrescens* and it's preyed-upon species is any indication of the potential for using other army ant predator-prey interactions.

## Note:

Bob Jeanne was editor of this paper
